# A Machine Learning Algorithm With an Oversampling Technique in Limited Data Scenarios for the Prediction of Present and Future Restorative Treatment Need: Development and Validation Study

**DOI:** 10.2196/75117

**Published:** 2025-08-28

**Authors:** Elina Väyrynen, Otso Tirkkonen, Henna Tiensuu, Jaakko Suutala, Vuokko Anttonen, Marja-Liisa Laitala, Katri Kukkola, Saujanya Karki

**Affiliations:** 1 Research Unit of Population Health Faculty of Medicine University of Oulu Oulu Finland; 2 Biomimetics and Intelligent Systems Group Faculty of Information Technology and Electrical Engineering University of Oulu Oulu Finland; 3 Oulu University Hospital Oulu Finland; 4 Optoelectronics and Measurement techniques Faculty of Information Technology and Electrical Engineering University of Oulu Oulu Finland

**Keywords:** oral health, machine learning, dentistry, dental caries, caries management

## Abstract

**Background:**

Untreated dental caries is the most common health condition worldwide. Therefore, new strategies need to be developed to reduce the manifestations of dental caries.

**Objective:**

This study aimed to develop and test a machine learning (ML) algorithm for detecting present and predicting future carious lesions in the adolescent population using a set of easy-to-collect predictive variables. In addition, this study aimed to deal with an imbalanced and small dataset using an oversampling method.

**Methods:**

This population-based study was conducted among secondary schoolchildren, aged between 13 and 17 years, from the northern parts of Finland in 2022. After meeting the inclusion criteria, a total of 218 participants were included in this study. The inclusion criteria consisted of participants having completed a web-based risk assessment questionnaire and having undergone a clinical examination at public health care services. Dental caries (International Caries Detection and Assessment System [ICDAS] scores of 4, 5, and 6; ie, ICDAS 4-6) and active initial caries (ICDAS 2+, 3+) were considered as outcomes. Several predictors, such as behavioral and dietary habits, were included. An extreme gradient boosting model was developed, tested, and assessed for its predictive performance. A 4-fold cross-validation was performed using the nested resampling technique. The random oversampling examples method and the k-nearest neighbors classifiers were used for all 4 folds. The mean (SD) performance of all the folds was computed.

**Results:**

Dental caries (ICDAS 2+,3+,4-6) were prevalent in 65.6% (143/218) of the participants. The mean area under the curve was 0.77 (SD 0.04) and the mean *F*_1_-score was 0.82 (SD 0.06) for the extreme gradient boosting model. Similarly, the mean area under the curve and mean *F*_1_-scores after oversampling were 0.74 (SD 0.05) and 0.79 (SD 0.04), respectively. The Shapley additive explanation values were calculated for all 4 folds to assess feature importance, revealing that previous dental fillings were the feature most strongly associated with the need for restorative treatment.

**Conclusions:**

On the basis of the performance metrics, the ML algorithm developed and tested in this study can be considered good. The ML algorithm could serve as a cost-effective screening tool for dental professionals to identify the risk of future restorative treatment needs. However, future studies with longitudinal cohorts and longitudinal data, along with external validation for generalizability, are needed to validate our model.

## Introduction

### Background

Dental caries is the most common dietary-microbial disease, requiring regular exposure to fermentable carbohydrates. Enrichment of acid-producing and acid-tolerating microorganisms in dental plaque leads to a demineralized tooth structure, which, in turn, can lead to loss of tooth structure, ultimately resulting in cavities [1]. The risks of dental caries include physical, biological, environmental, behavioral, and lifestyle-related factors [2]. The balance between pathological and protective factors, such as insufficient exposure to fluoride or irregular brushing of teeth, influences the initiation and progression of dental caries [3].

Individual-level risk recognition is of utmost importance, since recent dental caries management protocol prioritizes early prevention and minimal intervention at an individual level [4]. The availability of caries risk assessment tools (CRATs) has assisted clinicians in risk identification, as well as in risk minimization. However, most existing CRATs require either dental visits or measurements of salivary parameters at dental clinics (face-to-face methods). A systematic review [5] considered the possibility of using a reduced Cariogram (without saliva parameters), one of the CRATs, due to its better performance when compared to a full Cariogram. The use of artificial intelligence (AI) and machine learning (ML) in the medical field is gaining attention worldwide. The possibility of an automated dental caries risk prediction method using ML algorithms needs to be explored. A recent study by Xiong et al [6] highlighted the possibility of using ML algorithms and easy-to-collect predictors in screening active dental caries and urgent treatment needs in school-age children. However, the questionnaire mainly consisted of predictors covering physical, mental, and social aspects, missing important predictors, such as dietary habits and oral health-related behaviors. As the development and progression of carious lesions are multifactorial, such information is important to consider.

AI can be defined as the nonbiological ability of a computer to try to imitate human intelligence to accomplish complex tasks, such as problem-solving and decision-making [7]. ML is a subset of AI designed to identify patterns or make predictions based on the data used. ML algorithms can model nonlinear and high-dimensional characteristics, such as health data [8]. In addition to being the latest and often the most popular technology, ML algorithms have the ability to learn themselves and improve over time when exposed to more data [9]. The use of ML models can improve patient care by providing individualized outcome predictions and by reducing standardized processes, allowing clinicians to spend more time with patients [10]. In dentistry, the literature showed that various ML algorithms, such as logistic regression, decision trees, random forest, and extreme gradient boosting (XGBoost), are used in predicting dental caries. However, these studies are in their early stages, and more research needs to be conducted to validate these methods [11]. Furthermore, the ground truth in the above-mentioned studies is based only on clinical examinations (visual-tactile), even though it is recommended to perform both clinical and radiographic examinations to minimize the risk of misdiagnosis in caries evaluation [12]. In this regard, the severity and activity of carious lesions are crucial when deciding on the treatment path [13,14]. It is important to include real-life clinical environment observations when training and testing ML algorithms.

However, there are some challenges, such as high costs, data security, and legal restrictions in dentistry, making the acquisition of individual-level comprehensive data more difficult [15,16]. Studies have used electronic health records and national registries for training ML algorithms [17]. However, in dentistry, the availability of extensive electronic health record data or national registries is scarce [18]. Therefore, to overcome the challenges associated with small datasets, oversampling techniques can be explored. Oversampling is a data augmentation technique that aims to rebalance the training data distribution by amplifying the volume of instances that belong to the underrepresented class, helping to correct the imbalance between minority and majority examples [19,20]. Another challenge in dentistry is also related to class imbalance. When the number of patients with a target disease differs from the healthy population, the situation is referred to as the imbalanced data problem. The accuracy of ML models can be affected by these imbalances.

### Objectives

Therefore, this study aimed to (1) develop and test an ML algorithm in detecting present and predicting future carious lesions among adolescents using a set of easy-to-collect predictive variables; and (2) deal with challenges due to the imbalanced and small dataset in cariology with the use of the oversampling method.

## Methods

### Study Population and Data Sources

This cross-sectional study used data collected for the Digileap project, conducted among secondary school children aged 13 to 17 years from the northern parts of Finland in 2022. Before the study, the sample size (N=246) was calculated based on the prevalence of dental caries from a previous study by Suominen-Taipale et al [21] with 95% CIs and a precision set at 0.05, assuming that the total population of children aged 13 to 15 years is 100,000 [22]. Participants completed a web-based risk assessment questionnaire within their school premises, and their oral health records were registered at public health care services from 2022 to 2023, from where they were later requested through Findata services [23]. Findata is the Finnish Social and Health Data Permit authority, which grants permits for the secondary use of social and health care data, improving data protection for individuals. The inclusion criteria for this study included the following: (1) aged 13 to 17 years with signed informed consent, (2) completion of a web-based risk assessment questionnaire, and (3) completion of a dental examination performed at public dental clinics during 2022 to 2023. After meeting the inclusion criteria, a total of 218 participants were included in this study.

### Ethical Considerations

The study was conducted in full accordance with the World Medical Association Declaration of Helsinki. The ethical committee of Northern Ostrobothnia Hospital District approved the study protocol (EETTMK 62/2021), and the Finnish Medicines Agency [24] also issued the Medical Device Permit (2022/007715). In addition, study permissions were also obtained from the public health care services in Kuusamo, Ylivieska, Oulu, and Liminka. Oral health records (dental caries registered at the public dental services) were obtained from the Finnish Social and Health Data Permit Authority, Findata [25], with a data permit (THL/6268/14.02.00/2021)*.* All the schools were contacted before the study via an official email requesting the participation of schoolchildren and their parents. Participants aged ≥15 years signed the informed consent, and informed consent was obtained from the parents of participants aged <15 years. Participation was completely voluntary, privacy and confidentiality were secured, and the participants had the right to withdraw their participation at any given phase of the study.

### Study Variables

#### Outcome Variables

Initial active carious lesions (enamel and dentin caries) and all cavitated lesions were considered as the main outcome variable for this study. The carious lesions were diagnosed using the International Caries Detection and Assessment System (ICDAS) criteria with the aim to differentiate between different stages of dental caries. ICDAS stands for the assessment of the caries process by stage (noncavitated or cavitated) and activity (active or arrested or inactive). The “+” symbol indicates a caries lesion that is active and progressing. The “–” symbol indicates an inactive lesion with no active progression, and the tooth surface is considered sound [26].

To describe the transition of caries lesions in this study, the ICDAS score of 0 or ICDAS scores of 2− and 3− were considered as sound in contrast to the ICDAS scores of 2+, 3+, 4, 5, and 6 being considered as diseased. The ICDAS 2+ and 3+ codes were merged into 1 category (ICDAS 2+, 3+) to represent noncavitated lesions or microcavitated active lesions, and the ICDAS 4, 5, and 6 codes were used as 1 category (ICDAS 4-6) to represent cavitated lesions. A study by Abdalla et al [27] concluded that the active caries lesions were more likely to progress to more severe conditions than inactive lesions; active noncavitated (ICDAS 2+) and active microcavitated or shadow lesions (ICDAS 3+) had a 2-fold progression rate compared to noncavitated inactive lesions. In a nutshell, any initial active carious lesion (enamel and dentin caries) and all cavitated lesions were considered as the main outcome variable for this study (ICDAS 2+,3+,4-6) [13]. ICDAS 1 was not found in our study population due to challenges in diagnostics; these lesions were characterized by the first visual changes in enamel, often appearing as white spots or lines that were only visible when the tooth was carefully air-dried for 5 seconds.

Caries assessment with surface-by-surface evaluation was conducted by a licensed dentist following the Finnish Current Care Guidelines [28]. All teeth of the participants were examined with halogen light with a surface reflecting mirror and explorer and a fiberoptic transilluminator, followed by radiographic examination, if needed. A radiographic examination was suggested if (1) one localized enamel breakdown lesion was found, (2) the patient had several initial caries lesions, (3) the patient had dental caries risk factors or suspicion that the patient might have hidden dental caries lesions, or (4) radiographs had not been taken in the past few years [29]. Previously, data collected from the Finnish public health care records were shown not to be inferior to the calibrated examiners [30].

#### Predictors

For this study, age, sex, and oral health-related behaviors (such as frequency of toothbrushing, toothbrush type, toothpaste type, frequency of fluoride toothpaste use, interdental cleaning frequency, frequency of xylitol use, additive sugar consumption, and smoking habits) were considered as independent variables. The questionnaire consisted of information about the food and drink consumption of the participants. Participants were asked to report the amounts and frequencies. The average daily consumption was calculated for each product and multiplied by the quantity consumed. The additive sugar consumption was calculated using the Fineli database, a Finnish national food consumption database maintained by the Finnish Institute for Health and Welfare [31]. Using the Fineli database, total sugar content was matched to the food items, and the amount of sugar (g) in each item was calculated. Finally, the total daily sugar intake was calculated for each food item consumed per day (daily added sugar intake). Similarly, local factors, such as recent restorations, extracted teeth, bleeding when brushing, and the dry mouth index, were considered as predictors. To complement the self-reported survey, clinical data on missing teeth and dental fillings were also included as predictors. The dry mouth index included questions, such as “Does your mouth feel dry when you eat?” “Do you have difficulties swallowing some food?” and “Do you have to drink in order to make it easier to swallow dry food?” Participants responded yes or no, and answers were combined as one continuous variable, achieving values from 0 to 3.

#### Model Development and Training

In this study, the XGBoost algorithm was applied to predict the outcome variable. The model was trained using the R software (version 4.3.1; R Foundation for Statistical Computing) [32].

#### Model Fitting (Training and Testing)

**Figure 1:**
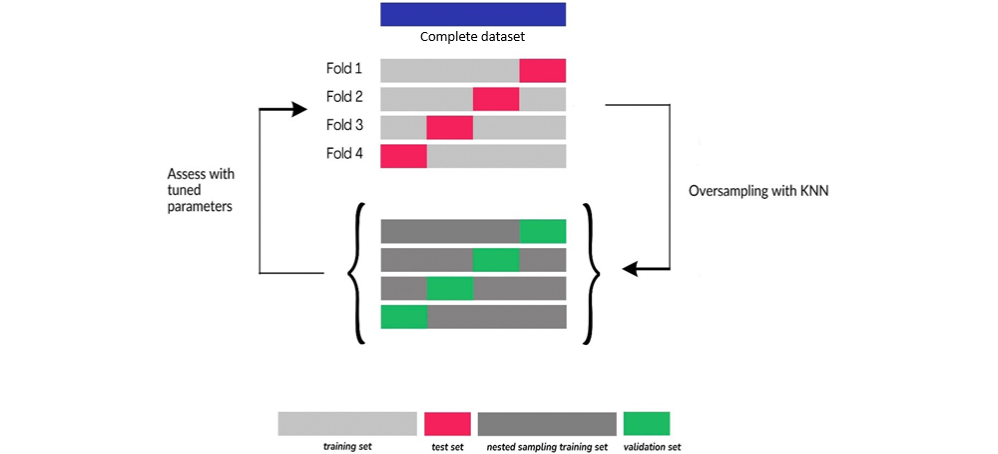
The model training protocol: The training and testing of machine learning algorithms were performed using 4-fold cross-validation, the nested resampling technique, and hyperparameter tuning. The tuned model was evaluated by a separate validation set, which was distinct from the nested sampling testing set. The oversampling technique was applied to all 4 folds. The oversampling fold included both the original training set and the nested sampling training set. Finally, the performance of the optimized model was assessed on each fold’s test set. This entire training protocol was repeated for all 4 folds. KNN: k-nearest neighbor.

The training and testing of the ML models were performed using the nested resampling technique with 4-fold cross-validation. In a typical k-fold cross-validation, the dataset is randomly and evenly split into k parts. The model is built using k−1 parts of the dataset, called the training set, and evaluated based on the remaining part, known as the test set. This process is repeated k times so that each part is used as a test set once [33]. Four folds were created, each containing 75% of the data as a training set and 25% of the unseen data as a test set. These 4 folds are seen in Figure 1.

#### Model Training Protocol

For each 4 folds, the ML models were built by using the mlr package [34]. During the training, hyperparameter tuning was conducted separately for all 4 folds. The grid search method was used for hyperparameter tuning, allowing the model to identify the optimal combination from a predefined set of hyperparameters. Detailed information on these predefined hyperparameters is provided in the Multimedia Appendix 1. The tuned model was evaluated based on a validation set, which was independent of the nested sampling testing set. Finally, the optimized model’s performances were evaluated on each fold’s completely unseen test set. For all 4 folds, the model training protocol was then repeated using the new oversampled training dataset. The model training protocol is shown in Figure 1.

#### Oversampling

As the total sample was 218, the oversampling technique was used as suggested by previous studies [35,36]. The oversampling technique was applied exclusively to each training dataset, while the respective test sets remained untouched to ensure the absence of data leakage. The random oversampling examples (ROSE) method and the k-nearest neighbors classifier were used for all folds [37]. Oversampling simulated 2000 new synthetic participants to the training dataset (*P*=.05). In the context of oversampling techniques, “P” refers to the proportion or percentage of the minority class instances that are to be oversampled. In the complete dataset, the number of participants with carious lesions was 143. The training sets consisted of 75% of the data, and the average number of participants with carious lesions in the training set was 0.75×143=107. Using the oversampling method, 2000×0.95=1900 new participants with carious lesions were created. As a result, the total number of participants with carious lesions increased on average to 107+1900=2007. Likewise, in the complete dataset, the number of participants with sound teeth was 75. The training set included 75% of the complete data, and the average number of participants with sound teeth in the training set was 0.75×75=56. Using the oversampling method, a total of 100 (2000×0.05=100) new synthetic participants with sound teeth were created. This resulted in an average total of 156 participants with sound teeth.

#### Model Evaluation

To assess the predictive performance of the ML models, the area under the curve (AUC), accuracy, sensitivity, specificity, positive predictive value, negative predictive value, no information rate, precision, recall, and *F*_1_-score for each predictive model were calculated. The mean (SD) performance of all folds was computed.

Shapley additive explanations (SHAP) values were computed for all folds and also after oversampling. The SHAP values were computed to determine the importance of each variable in predicting the dental caries outcomes of this study. The SHAP values are an additive feature importance measure that represents the responsibility of each feature in pushing the model output away from its base value [38]. This study was reported in accordance with the TRIPOD+AI (Transparent Reporting of a multivariable prediction model for Individual Prognosis Or Diagnosis–Artificial Intelligence) statement for developing or evaluating the performance of prediction models [39].

## Results

The demographic characteristics and descriptive analysis of the categorical independent variables are shown in Table 1. In this study group, 143 out of 218 (65.6%) participants had carious lesions (enamel and dental caries, ICDAS 2+, 3+, 4-6). More than half of the study participants were female (118/218, 54.1%), and more than half (124/218, 56.9%) had a habit of toothbrushing twice daily. Most of the participants (158/218, 72.5%) reported using fluoride toothpaste, while less than half of the participants (106/218, 48.6%) reported using an electronic toothbrush. Most of the participants (198/218, 90.8%) did not smoke, and the mean daily added sugar intake was 50.6 (SD 81.3) g. In addition, more than half of the participants (122/218, 56%) had dental restorations as seen in Table 1.

The performance metrics of all folds of the XGBoost model before and after oversampling are shown in Table 2. In addition, the mean performance across all folds is presented. The mean AUC value, which evaluates the model’s ability to discriminate between carious and sound teeth, was good after oversampling (before oversampling: 0.77, SD 0.04; after oversampling: 0.74, SD 0.05). The mean accuracy, which evaluates the performance of the models, was also high (before oversampling: 0.75, SD 0.06; after oversampling: 0.73, SD 0.03). The AUC and accuracy values were complemented by the *F*_1_-scores. The *F*_1_-score is the harmonic mean of precision and recall, and it provides a comprehensive evaluation of a model with an imbalanced dataset. The mean *F*_1_-score was 0.82 (SD 0.06) before oversampling and 0.79 (SD 0.04) after oversampling. The ability of the model to predict carious lesions (true positive cases), expressed as a mean sensitivity, was 0.85 (SD 0.12) before oversampling and 0.78 (SD 0.09) after oversampling. These values were considered high. The ability to predict sound teeth (true negative cases), expressed as mean specificity, was 0.56 (SD 0.13) before oversampling, and it slightly increased to 0.61 (SD 0.15) after oversampling. These values, in turn, were considered low, as seen in Table 2.

**Table 1:** Demographic characteristics and descriptive analysis of the categorical independent variables (N=218).

Characteristics			Values
**Fillings, n (%)**			
	No	96 (44)	
	Yes	122 (56)	
	Missing	0 (0)	
**Missing tooth, n (%)**			
	No	168 (77.1)	
	Yes	50 (22.9)	
	Missing	0 (0)	
**Smoking frequency, n (%)**			
	Not smoking	198 (90.8)	
	Smoking	20 (9.2)	
	Missing	0 (0)	
**Interdental cleaning frequency, n (%)**			
	At least twice a day	7 (3.2)	
	Once a day	18 (8.3)	
	2-6 times per week	47 (21.6)	
	Once a week	78 (35.8)	
	Never	68 (31.2)	
	Missing	0 (0)	
**Tooth extracted, n (%)**			
	No	174 (79.8)	
	Yes	44 (20.2)	
	Missing	0 (0)	
**Recent restoration, n (%)**			
	No	115 (52.8)	
	Yes	103 (47.2)	
	Missing	0 (0)	
**Bleeding while brushing, n (%)**			
	No bleeding	169 (77.5)	
	I do not know	26 (11.9)	
	Yes	23 (10.6)	
	Missing	0 (0)	
**Toothbrush type, n (%)**			
	Electric toothbrush	106 (48.6)	
	Variability both	48 (22)	
	Manual toothbrush	64 (29.4)	
	Missing	0 (0)	
**Toothbrushing frequency, n (%)**			
	At least twice a day	124 (56.9)	
	Once a day	67 (30.7)	
	2-6 times a week	22 (10.1)	
	Less often	5 (2.3)	
	Missing	0 (0)	
**Xylitol use frequency, n (%)**			
	Never	15 (6.9)	
	Once a month	9 (4.1)	
	1-3 times a month	15 (6.9)	
	Once a week	24 (11)	
	2-4 times a week	42 (19.3)	
	5-6 times a week	23 (10.6)	
	Once a day	17 (7.8)	
	2-3 times a day	48 (22)	
	>3 times a day	25 (11.5)	
	Missing	0 (0)	
**Toothpaste type, n (%)**			
	Fluoride	158 (72.5)	
	I do not know or fluoride-free	60 (27.5)	
	Missing	0 (0)	
**Fluoride paste use frequency, n (%)**			
	Daily	140 (64.2)	
	Few times a week	18 (8.3)	
	No or unsure of fluoride	60 (27.5)	
	Missing	0 (0)	
**Dry mouth index, n (%)**			
	0	104 (47.7)	
	1	51 (23.4)	
	2	9 (4.1)	
	Missing	54 (24.8)	
**Sex, n (%)**			
	Female	118 (54.1)	
	Male	100 (45.9)	
	Missing	0 (0)	
Age (y), mean (SD)			15.5 (1.11)
Daily added sugar intake^a^, mean (SD)			50.6 (81.3)
**Caries status, n (%)**			
	Healthy	64 (29.4)	
	Inactive enamel caries	11 (5)	
	Active enamel caries	125 (57.3)	
	Dentine caries	18 (8.3)	

^a^The sugars from each food item that the person consumed per day (g).

**Table 2:** Performance metrics of machine learning (ML) models before and after oversampling.

Fold and ML model		AUC^a^, mean (95% CI)	Accuracy, mean (95% CI)	NIR^b^	Sensitivity	Specificity	PPV^c^	NPV^d^	Precision	Recall	*F*_1_-score
**First fold**											
	XGBoost^e^	0.73 (0.59-0.87)	0.67 (0.53-0.79)	0.64	0.71	0.58	0.76	0.52	0.76	0.71	0.74
	XGBoost with oversampling	0.74 (0.56-0.88)	0.70 (0.56-0.82)	0.65	0.77	0.58	0.77	0.58	0.77	0.77	0.77
**Second fold**											
	XGBoost	0.82 (0.70-0.94)	0.80 (0.67-0.90)	0.62	0.97	0.52	0.77	0.92	0.77	0.97	0.86
	XGBoost with oversampling	0.80 (0.68-0.93)	0.76 (0.63-0.87)	0.62	0.79	0.71	0.82	0.68	0.82	0.79	0.81
**Third fold**											
	XGBoost	0.79 (0.66-0.92)	0.78 (0.65-0.88)	0.67	0.81	0.72	0.86	0.65	0.86	0.81	0.83
	XGBoost with oversampling	0.73 (0.59-0.87)	0.69 (0.55-0.81)	0.67	0.68	0.72	0.83	0.52	0.83	0.68	0.75
**Fourth fold**											
	XGBoost	0.74 (0.58-0.89)	0.76 (0.62-0.87)	0.69	0.92	0.41	0.77	0.70	0.77	0.92	0.84
	XGBoost with oversampling	0.70 (0.53-0.86)	0.74 (0.60-0.85)	0.69	0.89	0.41	0.77	0.64	0.77	0.89	0.83
**Performance across all folds^f^**											
	XGBoost	0.77 (0.04)	0.75 (0.06)	0.66 (0.03)	0.85 (0.12)	0.56 (0.13)	0.79 (0.05)	0.70 (0.16)	0.79 (0.04)	0.85 (0.10)	0.82 (0.06)
	XGBoost with oversampling	0.74 (0.05)	0.73 (0.03)	0.66 (0.03)	0.78 (0.09)	0.61 (0.15)	0.80 (0.54)	0.60 (0.07)	0.80 (0.03)	0.78 (0.08)	0.79 (0.04)

^a^AUC: area under the curve.

^b^NIR: no information rate.

^c^PPV: positive predictive value.

^d^NPV: negative predictive value.

^e^XGBoost: extreme gradient boosting.

^f^The performance across all folds is presented as mean (SD).

**Figure 2:**
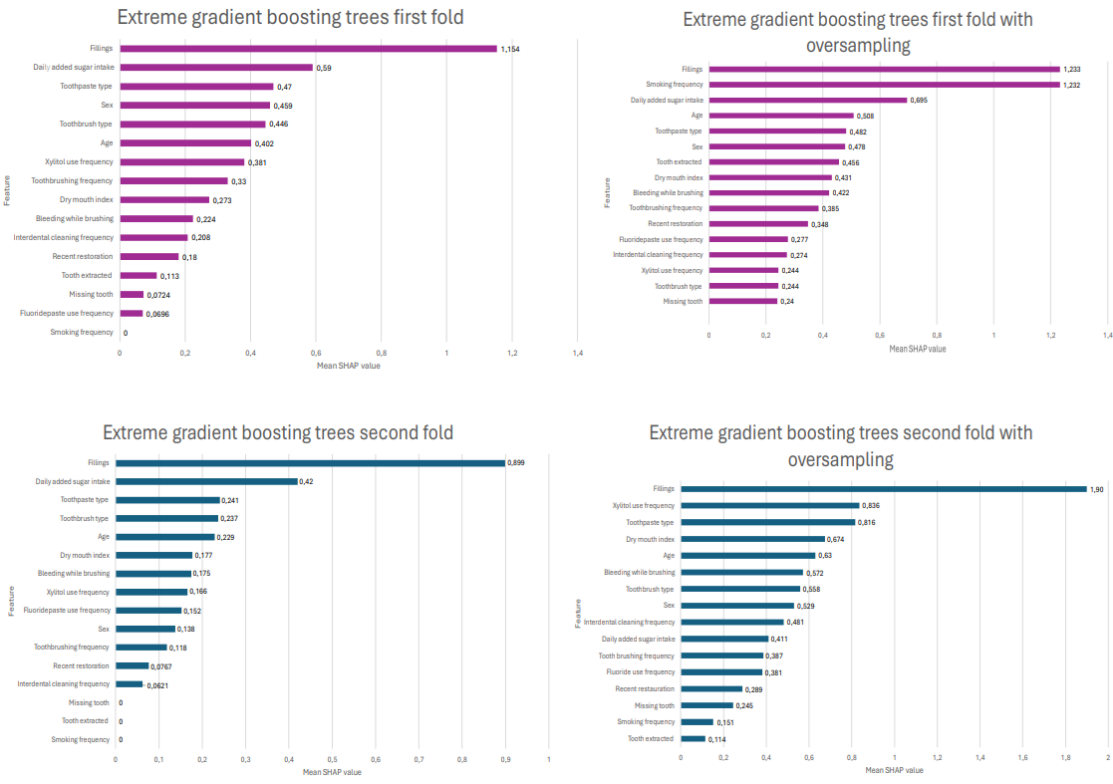
Shapley additive explanation (SHAP) values from folds 1 and 2, before and after oversampling. The absolute SHAP value shows how much a single feature affected the prediction of dental caries. The higher the SHAP value of a feature, the more likely it is to influence the prediction.

**Figure 3:**
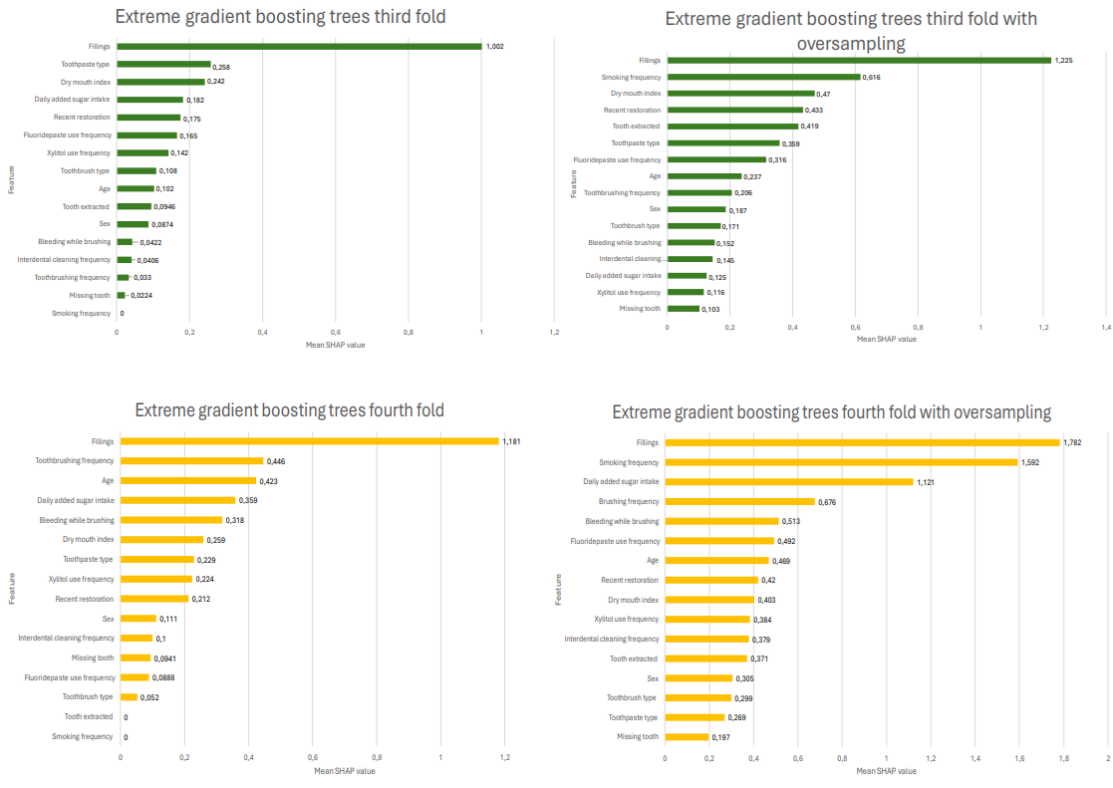
Shapley additive explanation (SHAP) values from folds 3 and 4, before and after oversampling. The absolute SHAP value shows how much a single feature affected the prediction of dental caries. The higher the SHAP value of a feature, the more likely it is to influence the prediction.

After evaluating the performance of each model, the SHAP values were computed for all 4 folds and after oversampling. The SHAP values for each of the 4 folds of the XGBoost model before and after oversampling are shown in Figures 2 and 3. The feature that most strongly predicted the need for present and future restorative treatment was previous fillings in all folds, followed by the total added sugar intake, frequency of smoking, toothpaste type, and frequency of toothbrushing, varying between 4 folds, as seen in Figures 2 and 3. Interestingly, the importance of minor predictors slightly increased after the oversampling method was applied in all folds. Fillings and total added sugar intake were in the top 4 most important features in every fold before oversampling. There was more variation in the folds after oversampling, but clearly, fillings remained the most important feature.

## Discussion

### Principal Findings

The main aim of this study was to develop and test an ML algorithm for predicting carious lesions among the adolescent population using a set of easy-to-collect predictors and to evaluate the importance of each predictor. Another aim was to use a novel oversampling approach in cariology research to deal with and improve the imbalance of a small dataset. The ML models developed and tested in this study performed well in predicting present and future restorative treatment needs among adolescents. Despite the drop in performance metrics after oversampling, the parameters were within the acceptable range, supporting the positive performance of the ML algorithm in this study. The XGBoost algorithm used in this study performed well. This is comparable with previous studies by Toledo et al [40] and Bomfim [41] from Brazil. In both studies, the XGBoost model outperformed other ML algorithms, such as logistic regression and decision tree. Both studies used socioeconomic variables, such as income and parents’ employment, as predictors for carious lesions. However, these variables were not considered in this study, because in Finland, all individuals aged ≤18 years are entitled to free dental care. This study aimed to use easy-to-collect predictors. A recent study by Xiong et al [6] also used ML algorithms and easy-to-collect information when screening active dental caries and urgent treatment needs in adolescents and concluded that the naïve Bayes model outperformed other models. However, that study particularly considered physical, mental, and social factors rather than behavioral factors. Furthermore, both clinical and radiographic examinations were performed in this study to minimize the risk of over- or underdiagnosis. In a real-life clinical environment, the use of radiographic methods is considered advantageous when deciding the need for operative care, especially when a patient was presented with an ICDAS score of 3 [29]. The drop in performance after oversampling in this study is comparable to a previous study [6] from the United States. Xiong et al [6] considered the synthetic minority oversampling technique for oversampling in their studies. However, the ROSE was used in this study for oversampling. The ROSE technique created synthetic examples by drawing from a smoothed bootstrap distribution in the feature space around the minority class, thus producing more balanced datasets with better generalization properties [42]. This method is particularly suitable for datasets containing categorical or binary variables, which were prominent in our study. In contrast, the synthetic minority oversampling technique was primarily designed for a continuous feature space and might not perform optimally with categorical or binary variables. In addition, the k-nearest neighbors classifier cleaning method was used in this study to further enhance the data quality after oversampling. In this study, the ability of the model to predict carious lesions (sensitivity) was high before and after oversampling. For dental caries screening, high sensitivity is vital to ensure that diseased individuals are correctly identified with the disease. However, the ability to predict sound teeth (specificity) was lower, which might be subject to overdiagnosis; therefore, cautious explanation is necessary. Therefore, oral health care professionals are encouraged to carefully examine those with high dental caries risk.

Dental caries is a multifactorial disease influenced by individual, biological, behavioral, and environmental factors [43]. In the literature, past caries experience was found to be the most powerful caries predictor [44]. High consumption of carbohydrates increased the chance of developing dental caries [45], and xylitol-containing products significantly prevented caries when compared with other nonxylitol products [46]. These findings from previous literature are in line with the results of this study, as delivered by the feature importance SHAP values. The past caries experience, nonuse of fluoridated toothpaste, socioeconomic level, and a higher frequency of sugar consumption were predictors that influenced caries progression the most in a previous longitudinal study that aimed to predict dental caries in primary and permanent teeth among children aged 1 to 5 years [40]. Similarly, the use of dental floss, unhealthy food consumption, self-declared race, and exposure to fluoridated water were the most predictive variables in another study by Bomfim [41]. The previous fillings (explaining past caries experience) and total added sugar intake (explaining high consumption of carbohydrates) were the most predictive variables in this study.

The application of SHAP values in this study enhanced the interpretability of the ML model, providing a transparent understanding of how each feature contributed to the predictions. Interpretability was a key element of explainable AI, which played a critical role in ensuring that the ML models were not only accurate but also transparent and reliable for real-world applications. In contrast to black-box models, explainable AI makes the model’s decisions more comprehensible and trustworthy. Understanding the rationale behind predictions helps in validating the model’s clinical relevance.

### Strengths and Limitations

One of the strengths of this study is that the outcome variable, carious lesions, was recorded by a licensed dentist based on both clinical and radiographic evaluations, following the Finnish Current Care Guidelines [29]. Another strength is the absence of missing values, which was ensured by the strict inclusion criteria of this study. Selection bias due to voluntary participation in this study can be considered a limitation. Another limitation is the potential for response bias due to the use of a self-reported questionnaire; self-reported data can be biased due to respondents’ subjective perceptions, memory recall issues, or intentional misreporting. However, the aim was to keep the questionnaire short and simple to minimize response bias. Finally, the generalizability of the ML algorithm might be questionable, as the model did not undergo external validation. Future studies with longitudinal cohorts are needed both to validate our models and to perform external validation in socioeconomically diverse or racially varied populations. In ML, validation provides evidence that a model is reliable and performs sufficiently with new data. External validation also requires testing the model on independent populations to assess its applicability [47]. Before clinical use, external validation is necessary [48]. However, this was beyond the scope of this study. For the external validation to be successful, dental caries categorization needs to be synchronized using ICDAS in both study populations.

### Clinical Implications

Rising health care costs associated with restorative treatment require justification in early prevention and control of dental caries. New strategies need to be developed to reduce social impacts, such as aesthetic and functional disturbances, on both the individual and societal levels. A potential application of ML algorithms in dental caries prognostic studies enables evidence‐based personalized dental care that could assist in decreasing dental caries prevalence globally. The ML model developed and tested in this study has the potential to identify possible risk factors of dental caries before the onset of actual dental caries lesions. In this study, each SHAP indicated the importance of each feature in dental caries progression, and the information gained can be transformed into a deeper dental caries risk assessment. Algorithm-based risk assessment tools can be integrated into electronic health records and used in electronic preassessment forms. Information about dental caries risk is valuable for both patients and dental professionals, influencing treatment and prevention plans, follow-up, and patient education [49]. Linking ML algorithms to intraoral images using deep learning algorithms is expected to increase dental screening potential. Individuals encounter unique challenges in adhering to behavioral changes. To overcome these obstacles, behavioral change interventions need to be both multifaceted and personalized. Health behavior factors, such as unhealthy food consumption, can be modified by health promotion policies and strategies. This study is unique and innovative because it is the first study to use ML models in dental caries prediction in adolescents using easy-to-collect predictors. In the future, after further development and external validation, this ML model could be used as a risk assessment tool and even be integrated into health record systems, which would be beneficial for the patient and the health care professionals in saving time and resources [50]. For the CRAT to be successful, it needs to be inexpensive, user-friendly, and open for everyone, even in low-income countries. SHAP values include participants’ dental caries risk profile and can be used for personalized behavioral change interventions in which patients themselves can alter their overall risk.

### Conclusions

Despite the small and imbalanced dataset, XGBoost performed well in predicting restorative treatment among adolescents with and without the oversampling method in this study. The results from this study suggest the potential feasibility of the ML models in caries risk assessment, enabling easier, cost-effective, less time-consuming, and more effective decision-making. However, future studies with longitudinal data and external validation are needed to validate our models.

## Appendix

Multimedia Appendix 1 Hyperparameter tuning using the grid search method.

## Abbreviations

AI: artificial intelligence
AUC: area under the curve
CRAT: caries risk assessment tool
ICDAS: International Caries Detection and Assessment System
ML: machine learning
ROSE: random oversampling examples
SHAP: Shapley additive explanations
TRIPOD+AI: Transparent Reporting of a multivariable prediction model for Individual Prognosis or Diagnosis–Artificial Intelligence
XGBoost: extreme gradient boosting

## Data Availability

Due to national legislation, restrictions apply to the availability of clinical data at the individual level, which were used with the Finnish Social and Health Data Permit Authority (Findata). Findata was responsible for the pseudonymization of the data and ensuring the anonymity of the results (THL/6268/14.02.00/2021).
