# Supplementary material for: A Machine Learning Algorithm With an Oversampling Technique in Limited Data Scenarios for the Prediction of Present and Future Restorative Treatment Need: Development and Validation Study
Source: JMIR Med Inform. 2025 Aug 28;13:e75117. doi: 10.2196/75117 (PMC12426571; doi:10.2196/75117)
Supplement: Multimedia Appendix 1 [file medinform_v13i1e75117_app1.docx]

Additional File 1. Hyperparameter tuning using Grid Search Method.

| **Hyperparameter** | **Values considered** |
| --- | --- |
| nrounds | 50, 100 |
| Max_depht | 3, 6 |
| eta | 0.1, 0.2, 0.3 |
| gamma | 0, 0.1, 0.2 |
| Colsample_bytree | 0.8 |
| Min_child_weight | 1, 3 |
| subsample | 0.7, 0.8 |
